# Identifying potentially low value surgical care: A national ecological study in England

**DOI:** 10.1177/13558196241252053

**Published:** 2024-05-09

**Authors:** Tim Jones, Angus McNair, Hugh McLeod, Josie Morley, Leila Rooshenas, William Hollingworth

**Affiliations:** 1 Research Fellow, The National Institute for Health Research Applied Research Collaboration West (NIHR ARC West), 1984 University Hospitals Bristol and Weston NHS Foundation Trust, Bristol, UK; 2 Research Fellow, Bristol Medical School, 1980 University of Bristol, Bristol, UK; 3 Associate Professor in Colorectal Surgery, Bristol Medical School, 1980 University of Bristol, Bristol, UK; 4 Consultant, North Bristol NHS Trust, Bristol, UK; 5 Senior Lecturer, NIHR ARC West, 1984 University Hospitals Bristol and Weston NHS Foundation Trust, Bristol, UK; 6 Senior Lecturer, Bristol Medical School, 1980 University of Bristol, Bristol, UK; 7 PhD Student, NIHR ARC West, 1984 University Hospitals Bristol and Weston NHS Foundation Trust, Bristol, UK; 8 PhD Student, Bristol Medical School, 1980 University of Bristol, Bristol, UK; 9 Associate Professor, Bristol Medical School, 1980 University of Bristol, Bristol, UK; 10 Professor, Bristol Medical School, 1980 University of Bristol, Bristol, UK

**Keywords:** applied health research, low value care, unwarranted variation

## Abstract

**Objectives:**

High variation in clinical practice may indicate uncertainty and potentially low-value care. Methods to identify low value care are often not well defined or transparent and can be time intensive. In this paper we explore the usefulness of variation analysis of routinely-collected data about surgical procedures in England to identify potentially low-value surgical care.

**Methods:**

This is a national ecological study using Hospital Episode Statistics linked to mid-year population estimates and indices of multiple deprivation in England, 2014/15-2018/19. We identified the top 5% of surgical procedures in terms of growth in standardised procedure rates for 2014/15 to 2018/19 and variation in procedure rates between clinical commissioning groups as measured by the systematic component of variance (SCV). A targeted literature review was conducted to explore the evidence for each of the identified techniques. Procedures without evidence of cost-effectiveness were viewed as of potentially low value.

**Results:**

We identified six surgical procedures that had a high growth rate of 37% or more over 5 years, and four with higher geographical variation (SCV >1.6). There was evidence for two of the 10 procedures that surgery was more cost-effective than non-surgical treatment albeit with uncertainty around optimal surgical technique. The evidence base for eight procedures was less clear cut, with uncertainty around clinical- and/or cost-effectiveness. These were: deep brain stimulation; removing the prostate; surgical spine procedures; a procedure to alleviate pain in the spine; surgery for dislocated joints due to trauma and associated surgery for traumatic fractures; hip joint replacement with cemented pelvic component or cemented femoral component; and shoulder joint replacement.

**Conclusions:**

This study demonstrates that variation analysis could be regularly used to identify potentially low-value procedures. This can provide important insights into optimising services and the potential de-adoption of costly interventions and treatments that do not benefit patients and the health system more widely. Early identification of potentially low value care can inform prioritisation of clinical trials to generate evidence on effectiveness and cost-effectiveness before treatments become established in clinical practice.

## Introduction

Health systems globally are striving to optimise the delivery of health services and there is increasing awareness of the role of ‘low value’ care in this process. In the field of surgery, there are concerns that some surgical procedures may not be effective or cost-effective for some or all patients in whom they are used. For example, recent placebo-controlled randomised trials have indicated that subacromial decompression, a commonly-used surgery for shoulder pain, is no more effective than investigational arthroscopy.^1,2^ Procedures such as subacromial decompression are thus considered low value because they are unlikely to benefit patients given the potential harms, costs, or patients’ preferences.^3^

It is against this background that there has been a shift towards de-adopting existing low value treatments.^4,5^ One example is the Choosing Wisely initiative, which began in the USA and has since been implemented in other countries.^6^ Choosing Wisely is a physician-led approach, which involves medical specialty societies identifying tests and procedures for which there is evidence of overuse or harm. In England, the National Health Service (NHS) Evidence Based Interventions (EBI) programme has identified procedures to be de-adopted since 2019, with decisions informed by National Institute for Health and Care Excellence (NICE) guidelines, international recommendations, Choosing Wisely lists, and geographic variation, although how exactly de-adopting decisions are being arrived at is not clear.^7^

Initiatives to de-adopt existing technologies and procedures face considerable challenges.^8–10^ Key among these is the methodological approach that would allow for identifying technologies and procedures where there is uncertainty about their value. One such approach is to use existing data to explore geographic variation of the use of a given procedure. This approach draws on the professional uncertainty hypothesis postulated by Wennberg and colleagues.^11,12^ It suggests that where the evidence that the benefits of a procedure outweigh the risks/costs is inconclusive, practitioners’ (e.g., surgeons) beliefs and preferences will determine local practice patterns rather than clinical need, and this will, over time, be observable as variation in practices (e.g. procedure rates) between sites, such as hospitals or small areas. Measurement of geographic variation can therefore provide important insight into procedures of uncertain value, while measurement of variation over time (e.g. by year) can usefully identify procedures that are increasingly being used because of or in spite of emerging evidence. In this paper, we used these approaches to explore geographic and temporal variation in surgical procedure rates in hospitals in England. Our aim was to identify procedures with the highest geographic variation and 5-year growth rate. We also conducted targeted evidence reviews on the clinical- and cost-effectiveness of these procedures.

## Methods

This was a longitudinal observational study using routinely collected administrative data about patients admitted to hospitals in England for surgery for the period 2014/15 to 2018/19. We followed the RECORD extension to STROBE guidelines for observational studies.^13^

### Data sources

Hospital procedures were identified using ‘admitted patient care’ hospital episode statistics (HES-APC) data. HES-APC is a routinely collected dataset that records all episodes of admitted (day case or inpatient) care provided to patients at NHS hospitals in England and to NHS-funded patients treated in private hospitals.^14^ Each episode represents a period of care under one consultant team. Up to 20 diagnoses are recorded per episode using the International Classification of Diseases (ICD) version 10. Up to 24 clinical procedures per episode may be recorded using Office of Population, Censuses and Surveys (OPCS) (fourth revision) procedure codes. The first recorded diagnosis and the first recorded procedure are considered the ‘primary’ diagnosis and ‘most resource intensive’ procedure for that episode. HES also includes the Lower Super Output Area (LSOA; an area of around 1500 people) of residence for each patient.

We used mid-year population estimates by age, sex, and LSOA from the Office for National Statistics to estimate procedure rates.^15^ Area-level ethnicity by LSOA was gathered from the Census of England and Wales, 2011.^16^ Deprivation by LSOA was quantified using the English Indices of Multiple Deprivation (2015).^17^ Lookup tables from LSOA to clinical commissioning groups (CCGs; managed NHS care in local areas of England until 2022, covering around 225,000 people) were used to aggregate our analyses. Average procedure costs were estimated from 2018/19 NHS tariffs^18^ linked to health care resource group (HRG) codes and length of stay for each admission.

### Included procedures

We included 3-character OPCS-4 procedure codes recorded as the primary procedure in >= 1350 hospital admissions in 2018/19. This threshold is equivalent to 10 admissions on average in each of the 135 clinical commissioning groups (CCGs) in England, which is recommended for robust directly standardised rates.^19^ Procedures were placed into three categories:^20^ *inclusive* (minor surgery, interventional radiology procedures and diagnostic endoscopies), *intermediate* (procedures routinely undertaken in an operating theatre and/or under general or regional anaesthesia), and *restrictive* (surgery that due to duration or complexity often results in tissue injury). We excluded procedures categorised as non-surgical by Abbott et al.^20^ This study focussed on restrictive procedures only because they are typically more complex and higher cost. Procedure codes in each category are provided in Table S1 (Online supplement).

### Identification of procedures with high geographic variation and growth

Procedures were counted once for each time they were listed as primary procedure in a continuous hospital spell.^21^ Age-sex standardised rates^22^ were calculated per 100,000 population using mid-year populations for each financial year. The yearly national age-sex specific rates were applied to the age-sex specific population of each CCG to calculate expected procedure counts for that area. CCG rates were further adjusted for ethnicity and deprivation by including these variables in a Poisson model of observed counts with logged expected counts as the offset. The predicted counts from the Poisson model became the adjusted expected counts, the Poisson model having essentially adjusted for variation due to ethnicity and deprivation. In a sensitivity analysis we repeated the age-sex standardisation of procedure rates without further adjustment for ethnicity and deprivation.

Growth in procedure rates over time was estimated by dividing the 2018/19 rate by the 2014/15 rate. Geographical variation in procedure rates by CCG in 2018/19 was estimated using the systematic component of variance (SCV),^23,24^ which indicates the amount of variation between CCGs after adjusting for chance variation (see Online supplement for further detail). When referring to geographical variation, we mean as measured by the SCV between CCGs.

We identified restricted procedures that were in the top 5% in terms of geographic variation or growth; they were examined at the 4-character OPCS level to explore homogeneity within 3-character codes. Similarly, homogeneity of primary diagnosis was explored by summarising ICD-10 codes corresponding to selected procedures. Procedures with high variation were mapped using ArcMap 10.7.1.

### Targeted evidence reviews for identified procedures

We conducted targeted reviews of the evidence for identified procedures. This focused on national clinical guidelines identified from the NICE database,^25^ systematic reviews identified from the Cochrane database,^26^ and recent or ongoing systematic reviews, randomised trials, or economic studies supported by the National Institute for Health and Care Research (NIHR).^27^ We further searched Medline for economic studies on each selected procedure (Supplemental Table S2). One author [TJ] screened Medline titles and abstracts using Rayyan software^28^ to identify economic studies carried out alongside RCTs for each topic.

Using evidence obtained from systematic reviews, economic studies, NICE guidelines, or ongoing NIHR-funded studies, procedures were grouped into those where: (i) published evidence indicates surgery is effective but there is uncertainty about the optimal operative technique and (ii) there is uncertain (cost-)effectiveness. A brief summary is presented to illustrate the evidence. Procedures without evidence of cost-effectiveness were viewed as of potentially low value.

## Results

We included a total of 134 restrictive 3-character procedure codes in our analysis, with a median procedure rate in 2018/19 of 7.13 per 100,000 population (IQR: 4.19, 16.55). The average growth in procedure rates in the 5 years from 2014/15 to 2018/19 was 0.98 (SD: 0.22), and median systematic component of variance in 2018/19 was 0.14 (IQR: 0.05, 0.32).

### Procedures with high geographic variation and growth

**Figure 1.**
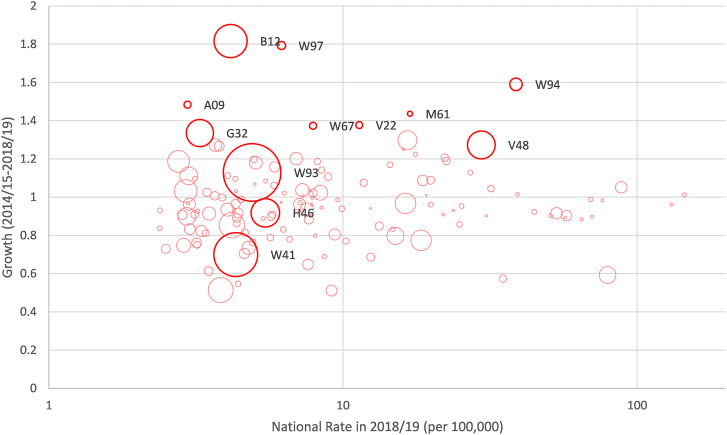
National procedure rates (2018/19), growth in procedure rates (2014/15-2018/19), and geographic variation (SCV) in procedure rates (2018/19) for restrictive procedures. *Note*: Larger bubble size is proportional to greater geographic variance (SCV) in procedure rates. When growth is <1 the procedure rate has reduced over the 5 years. Highlighted (bold) circles are either the top 5% in terms of growth (>1.37) or geographic variance (SCV) in 2018/19 (>1.59). Procedure codes: B12 – Other operations on thyroid gland; W97 - Shoulder joint replacement; A09 – Deep brain stimulation for involuntary posturing; W94 – Hip joint replacement (cemented femoral component); G32 – Bariatric bypass surgical procedure; W67 – Joint surgery for traumatic dislocation and fracture; V22 – Surgical spine procedures; M61 – Removal of prostate; V48 – Procedure to alleviate pain in the spine; W93 – Hip joint replacement (cemented pelvic component); H46 - Other operations on rectum; W41 - Total knee joint replacement (uncemented).

Figure 1 plots all restrictive surgical procedures in terms of their national procedure rate in 2018/19 (per 100,000 population), growth in procedure rate (between 2014/15 and 2018/19), and geographical variation (SCV) in 2018/19. We identified 12 restrictive procedures as high growth or having a high degree of geographical variation by CCG (SCV).

Of these, two procedures were of predominantly diagnostic nature (biopsy of lesion of thyroid (B12.2); intubation of rectum for pressure manometry (H46.3)) and excluded from further analyses. Of the remaining 10 procedures, four were highlighted because of high geographical variation by CCG (SCV) and six due to high growth (Table 1). The procedure with the highest growth was shoulder joint replacement, which rose from 3.44 procedures per 100,000 population in 2014/15 to 6.18 per 100,000 in 2018/19 (79%). The procedure with the highest variation by CCG in 2018/19 was hip joint replacement with cemented pelvic component with an SCV of 8.05; six CCGs recorded none of these procedures for their population, while three CCGs recorded over 60 per 100,000 population.

**Table 1.** Restrictive procedures with high increase in procedure rates (2014/15-2018/19) and/or high geographical variation in 2018/19.

Procedure summary descriptors	Procedure code	National rate per 100,000 (2018/19)	Growth (2014/15 - 2018/19)	Variation by CCG (SCV 2018/19)	5^th^ percentile rate per 100,000 (2018/19)	95^th^ percentile rate per 100,000 (2018/19)
*Procedures in the top 5% for high variation*						
Bariatric bypass surgical procedure	G32	3.26	1.34	1.88	0	10.45
Procedure to alleviate pain in the spine	V48	29.52	1.27	1.96	4.99	119.06
Total knee joint replacement (uncemented)	W41	4.32	0.70	4.75	0.42	12.11
Hip joint replacement (cemented pelvic component)	W93	4.91	1.13	8.05	0.13	40.26
*Procedures in the top 5% for high growth*						
Deep brain stimulation for involuntary posturing	A09	2.96	1.48	0.16	0.53	5.17
Removal of prostate	M61	16.88	1.44	0.09	6.85	24.59
Surgical spine procedures	V22	11.35	1.38	0.15	4.87	19.95
Joint surgery for traumatic dislocation and fracture	W67	7.90	1.37	0.15	2.88	14.11
Hip joint replacement (cemented femoral component)	W94	38.73	1.59	0.44	6.69	94.14
Shoulder joint replacement	W97	6.18	1.79	0.20	1.88	11.87

Note: See Table S3 for full procedure descriptions. Growth is the standardised procedure rate in 2018/19 divided by the standardised procedure rate in 2014/15. SCV is Systematic Component of Variance. We provide the 95^th^ percentile standardised procedure rate and the 5^th^ percentile standardised procedure rate by CCG.

There was strong regional clustering of procedures. For example, the use of the bariatric bypass surgical procedure was highest in the South East and North East of England, while the use of total knee joint replacement (uncemented) was highest in the West and South Midlands, but low in the South West and far North (see Supplemental Figures S1-S4).

The vast majority of each of the 10 identified procedures were carried out electively, with the lowest being joint surgery for traumatic dislocation and fracture, for which 78% were carried out electively. Supplemental Table S3 shows that some procedures were carried out largely for a single condition: stomach bypass for obesity (92%); removal of prostate for prostate cancer (97%); knee replacement for arthrosis of the knee (85%); and hip replacement for arthrosis of the hip (76%–79%). The other procedures were carried out for a more diverse range of conditions.

We estimated the total costs of the procedures identified as high growth or with high variation by CCG. In 2018/19, the cost ranged from £2.9 million for deep brain stimulation to £58.9 million for hip joint replacement using cemented femoral component. The sensitivity analysis using age-sex standardisation of procedure rates without adjustment for deprivation and ethnicity identified nine of the same procedures but included ‘Other connection of stomach to jejunum’ (G33) instead of ‘Connection of stomach to transposed jejunum’ (G32). These are alternative surgical techniques for obesity and this did not alter the findings.

### Evidence base for identified procedures

Our targeted review of the evidence base found some support, in terms of cost-effectiveness compared to non-surgical techniques, for two of the four procedures with high geographic variation (bariatric surgical procedure and total knee replacement, uncemented), although there remains uncertainty around the optimal surgical technique (Supplemental Table S4). We identified six procedures with high growth over five years. For two of these procedures (deep brain stimulation for involuntary posturing and open removal of prostate) there was only very limited evidence of cost-effectiveness (Supplemental Table S4). There was no evidence of cost-effectiveness for the other four procedures (Supplemental Table S4).

The absence of evidence about the cost-effectiveness of eight of the 10 included procedures suggests that the methods used can play a useful role in identifying potentially low value surgical care.

## Discussion

This study demonstrates that relatively simple methods can be used to highlight surgical procedures that are potentially of low value. We identified four procedures that had high geographical variation. For two of these there was evidence that surgery was effective and cost-effective, and variation was largely due to unclear evidence about the most appropriate surgical technique (obesity surgery and hip/knee replacements). Six procedures had large increases in use over five years. The evidence of effectiveness or cost-effectiveness for all these procedures was mixed, unclear or incomplete. In most cases, uncertainty about effectiveness and cost-effectiveness has been recognised, and clinical trials are ongoing.

### Strengths and weaknesses of the study

Our study used a national, longitudinal dataset over a five-year period covering all NHS secondary care providers in England. The HES data is used for determining payments for hospitals, which provides a strong incentive to record procedures. However, accuracy and completeness of data coding, as well as clinical care, is likely to vary between hospitals and individuals, which may account for some of the variation in our results. Population denominators, and linkage to the indices of multiple deprivation and ethnicity data, allowed us to investigate trends and variations in procedure rates standardised on age, sex, deprivation and ethnicity. There may be other factors influencing rates which we have not controlled for. For example, literacy and language barriers might influence access to care; also, the centralisation of specialised services could mean that distance to these services influences their use and impacts on variation. By design, our method is unable to account for every possible reason for variation. However, we provide a simple approach to identify surgical procedures where rates are rapidly increasing or vary between localities for further detailed exploration.

For simplicity and practicality, our analysis used 3-character OPCS-4 procedure codes which rarely map uniquely onto clinical procedures. Surgical procedures can potentially be coded in different ways, requiring a combination of OPCS procedure codes and ICD-10 diagnosis codes to be accurately identified. While coding algorithms for specific procedures have been published, we are not aware of a standardised way of identifying a large range of surgical procedures using OPCS and ICD codes.

Our literature review was not a full systematic review, and this was not the aim of this study. We believe that our targeted approach enabled us to identify major evidence available within a reasonable timeframe, although we accept we may have missed some relevant evidence.

### Strengths and weaknesses in relation to other similar studies

The procedure to alleviate pain in the spine was also identified by the NHS England EBI programme as lacking evidence of cost-effectiveness. We have focused on more expensive and complex surgery procedures in the top 5% in terms of growth or variation. Relaxing these criteria might have resulted in more overlap with potentially low value procedures that were also identified by the EBI programme. Our method offers a complementary opportunity to identify surgical procedures where use is rapidly changing or highly variable.

### Implications for policymakers and clinicians

Our study identified a number of surgical procedures that are currently used in the NHS that may be of low value. A simple variation analysis such as that used in this study could be carried out on a regular basis to identify other potentially low-value procedures, which could then be investigated in more detail. Such an approach can usefully inform the optimisation of service delivery through de-adoption of costly interventions and treatments that do not benefit patients. It can also inform prioritisation of research funding to generate a sufficiently robust evidence based on (cost-)effectiveness before potentially low value procedures become established in practice. Such an approach would complement the existing national programme to promote local assessment of potentially unwarranted variation.^29^

### Unanswered questions and future research

While our work has identified eight potential candidate surgical procedures that may be of low value that warrant further investigation for potential de-adoption, our literature review was not exhaustive. It is rare to find a procedure that is of low value for all patients and therefore interventions should be limited to those who would benefit most. Evidence often lacks certainty about the effectiveness, costs, and safety within population subgroups and it is often not possible to draw firm conclusions on what clinical criteria to use to guide use of surgery.^8,30^ A higher evidential threshold may be required to justify the reduction in use of an existing technology compared to adoption of a new one.^31^ Further work could explore the use of and evidence for these procedures, including qualitative exploration with clinicians, commissioners and patients regarding the use of these techniques, the acceptability of their potential de-adoption, and how de-adoption might work in practice.^32^

## Supplemental Material

Supplemental Material - Identifying potentially low value surgical care: A national ecological study in England Supplemental Material for The surgeons’ cut: Identifying potentially low value surgical care – an English national ecological study by Tim Jones, Angus McNair, Hugh McLeod, Josie Morley, Leila Rooshenas and William Hollingworth in Journal of Health Services Research & Policy
