# Supplementary material for: Identifying potentially low value surgical care: A national ecological study in England
Source: J Health Serv Res Policy. 2024 May 9;29(4):223–9. doi: 10.1177/13558196241252053 (PMC11346124; doi:10.1177/13558196241252053)
Supplement: Supplemental Material - Identifying potentially low value surgical care: A national ecological study in England [file sj-pdf-1-hsr-10.1177_13558196241252053.pdf]

Contents

Table S1: Procedures included in the study and their 'Abbott' category of surgical complexity ... 2

Table S2. Search strategies used for targeted literature reviews for the 10 identified procedures 13

Table S3. The most common primary procedures and primary diagnoses recorded for each of the 10 included ‘restrictive’ procedures. 18

Table S4. Evidence of clinical and cost-effectiveness relating to highlighted procedures and related NICE guidance 20

Figure S1. Variation in standardised rates of connection of stomach to transposed jejunum (G32) by CCG in 2018/19 26

Figure S2. Variation in standardised rates of denervation of spinal facet joint of vertebra (V48) by CCG in 2018/19 27

Figure S3. Variation in standardised rates of total prosthetic replacement of knee joint not using cement (W41) by CCG in 2018/19 28

Figure S4. Variation in standardised rates of hybrid prosthetic replacement of hip joint using cemented acetabular component (W93) by CCG in 2018/19 29

Figure S5. National procedure rates (2018/19), growth in procedure rates (2014/15-2018/19), and geographic variation in procedure rates (2018/19) for ‘inclusive’ procedures. 30

Figure S6. National procedure rates (2018/19), growth in procedure rates (2014/15-2018/19), and geographic variation in procedure rates (2018/19) for ‘intermediate’ procedures. 31

Methods S1. Systematic component of variance. 32

Table S1: Procedures included in the study and their 'Abbott' category of surgical complexity

Procedure Code	Procedure Description	Abbott Category
A02	Excision of lesion of tissue of brain	Restrictive
A09	Neurostimulation of brain	Restrictive
A12	Creation of connection from ventricle of brain	Restrictive
A41	Drainage of subdural space	Restrictive
A48	Other operations on spinal cord	Restrictive
A57	Operations on spinal nerve root	Restrictive
B08	Excision of thyroid gland	Restrictive
B12	Other operations on thyroid gland	Restrictive
B14	Excision of parathyroid gland	Restrictive
B27	Total excision of breast	Restrictive
B28	Other excision of breast	Restrictive
B29	Reconstruction of breast	Restrictive
D10	Exenteration of mastoid air cells	Restrictive
E13	Other operations on maxillary antrum	Restrictive
E42	Exteriorisation of trachea	Restrictive
E54	Excision of lung	Restrictive
E55	Open extirpation of lesion of lung	Restrictive
E59	Other operations on lung	Restrictive
F23	Extirpation of lesion of tongue	Restrictive
F28	Extirpation of lesion of palate	Restrictive
F38	Extirpation of lesion of other part of mouth	Restrictive
G01	Excision of oesophagus and stomach	Restrictive
G21	Other operations on oesophagus	Restrictive
G23	Repair of diaphragmatic hernia	Restrictive
G24	Antireflux operations	Restrictive
G28	Partial excision of stomach	Restrictive
G30	Plastic operations on stomach	Restrictive
G32	Connection of stomach to transposed jejunum	Restrictive
G33	Other connection of stomach to jejunum	Restrictive
G34	Artificial opening into stomach	Restrictive
G69	Excision of ileum	Restrictive
H01	Emergency excision of appendix	Restrictive
H02	Other excision of appendix	Restrictive
H06	Extended excision of right hemicolon	Restrictive
H07	Other excision of right hemicolon	Restrictive
H10	Excision of sigmoid colon	Restrictive
H15	Other exteriorisation of colon	Restrictive
H30	Other operations on colon	Restrictive
H33	Excision of rectum	Restrictive
H41	Other operations on rectum through anus	Restrictive
H46	Other operations on rectum	Restrictive
H62	Other operations on bowel	Restrictive

Procedure Code	Procedure Description	Abbott Category
J02	Partial excision of liver	Restrictive
J18	Excision of gall bladder	Restrictive
K25	Plastic repair of mitral valve	Restrictive
K26	Plastic repair of aortic valve	Restrictive
K40	Saphenous vein graft replacement of coronary artery	Restrictive
K45	Connection of thoracic artery to coronary artery	Restrictive
K57	Other therapeutic transluminal operations on heart	Restrictive
L19	Other replacement of aneurysmal segment of aorta	Restrictive
L27	Transluminal insertion of stent graft for aneurysmal segment of aorta	Restrictive
L29	Reconstruction of carotid artery	Restrictive
L59	Other bypass of femoral artery	Restrictive
L60	Reconstruction of femoral artery	Restrictive
L67	Excision of other artery	Restrictive
L70	Other open operations on other artery	Restrictive
L74	Arteriovenous shunt	Restrictive
L75	Other arteriovenous operations	Restrictive
L79	Other operations on vena cava	Restrictive
M01	Transplantation of kidney	Restrictive
M02	Total excision of kidney	Restrictive
M03	Partial excision of kidney	Restrictive
M34	Total excision of bladder	Restrictive
M61	Open excision of prostate	Restrictive
M73	Repair of urethra	Restrictive
N06	Other excision of testis	Restrictive
O17	Secondary closed reduction of fracture of bone and internal fixation	Restrictive
O27	Other stabilising operations on joint	Restrictive
O29	Excision of bone	Restrictive
P05	Excision of vulva	Restrictive
P20	Extirpation of lesion of vagina	Restrictive
Q01	Excision of cervix uteri	Restrictive
Q07	Abdominal excision of uterus	Restrictive
Q08	Vaginal excision of uterus	Restrictive
Q22	Bilateral excision of adnexa of uterus	Restrictive
Q23	Unilateral excision of adnexa of uterus	Restrictive
Q43	Partial excision of ovary	Restrictive
S55	Exploration of burnt skin of other site	Restrictive
T10	Therapeutic endoscopic operations on pleura	Restrictive
T36	Operations on omentum	Restrictive
T41	Other open operations on peritoneum	Restrictive
T52	Excision of other fascia	Restrictive
T77	Excision of muscle	Restrictive
T85	Block dissection of lymph nodes	Restrictive
V09	Reduction of fracture of other bone of face	Restrictive

**Procedure Code**	**Procedure Description**	**Abbott Category**
V10	Division of bone of face	Restrictive
V15	Reduction of fracture of mandible	Restrictive
V17	Fixation of mandible	Restrictive
V22	Primary decompression operations on cervical spine	Restrictive
V25	Primary decompression operations on lumbar spine	Restrictive
V26	Revisional decompression operations on lumbar spine	Restrictive
V29	Primary excision of cervical intervertebral disc	Restrictive
V33	Primary excision of lumbar intervertebral disc	Restrictive
V38	Primary fusion of other joint of spine	Restrictive
V40	Stabilisation of spine	Restrictive
V41	Instrumental correction of deformity of spine	Restrictive
V44	Decompression of fracture of spine	Restrictive
V48	Denervation of spinal facet joint of vertebra	Restrictive
V54	Other operations on spine	Restrictive
V67	Other primary decompression operations on lumbar spine	Restrictive
W06	Total excision of bone	Restrictive
W08	Other excision of bone	Restrictive
W09	Extirpation of lesion of bone	Restrictive
W15	Division of bone of foot	Restrictive
W16	Other division of bone	Restrictive
W19	Primary open reduction of fracture of bone and intramedullary fixation	Restrictive
W20	Primary open reduction of fracture of bone and extramedullary fixation	Restrictive
W21	Primary open reduction of intra-articular fracture of bone	Restrictive
W23	Secondary open reduction of fracture of bone	Restrictive
W24	Closed reduction of fracture of bone and internal fixation	Restrictive
W25	Closed reduction of fracture of bone and external fixation	Restrictive
W28	Other internal fixation of bone	Restrictive
W30	Other external fixation of bone	Restrictive
W37	Total prosthetic replacement of hip joint using cement	Restrictive
W38	Total prosthetic replacement of hip joint not using cement	Restrictive
W39	Other total prosthetic replacement of hip joint	Restrictive
W40	Total prosthetic replacement of knee joint using cement	Restrictive
W41	Total prosthetic replacement of knee joint not using cement	Restrictive
W42	Other total prosthetic replacement of knee joint	Restrictive
W46	Prosthetic replacement of head of femur using cement	Restrictive
W47	Prosthetic replacement of head of femur not using cement	Restrictive
W57	Excision reconstruction of joint	Restrictive
W58	Other reconstruction of joint	Restrictive
W62	Other primary fusion of other joint	Restrictive
W65	Primary open reduction of traumatic dislocation of joint	Restrictive
W67	Secondary reduction of traumatic dislocation of joint	Restrictive
W80	Debridement and irrigation of joint	Restrictive
W93	Hybrid prosthetic replacement of hip joint using cemented acetabular	Restrictive

Procedure Code	Procedure Description	Abbott Category
	component	
W94	Hybrid prosthetic replacement of hip joint using cemented femoral component	Restrictive
W96	Total prosthetic replacement of shoulder joint using cement	Restrictive
W97	Total prosthetic replacement of shoulder joint not using cement	Restrictive
X08	Amputation of hand	Restrictive
X09	Amputation of leg	Restrictive
X21	Correction of congenital deformity of hand	Restrictive
A60	Destruction of peripheral nerve	Intermediate
A61	Extirpation of lesion of peripheral nerve	Intermediate
A62	Microsurgical repair of peripheral nerve	Intermediate
A64	Other repair of peripheral nerve	Intermediate
A65	Release of entrapment of peripheral nerve at wrist	Intermediate
A67	Release of entrapment of peripheral nerve at other site	Intermediate
A69	Revision of release of peripheral nerve	Intermediate
A70	Neurostimulation of peripheral nerve	Intermediate
A73	Other operations on peripheral nerve	Intermediate
A81	Other operations on sympathetic nerve	Intermediate
A84	Neurophysiological operations	Intermediate
B30	Prosthesis for breast	Intermediate
B31	Other plastic operations on breast	Intermediate
B33	Incision of breast	Intermediate
B34	Operations on duct of breast	Intermediate
B36	Reconstruction of nipple and areola	Intermediate
B37	Other operations on breast	Intermediate
C10	Operations on eyebrow	Intermediate
C11	Operations on canthus	Intermediate
C12	Extirpation of lesion of eyelid	Intermediate
C13	Excision of redundant skin of eyelid	Intermediate
C14	Reconstruction of eyelid	Intermediate
C15	Correction of deformity of eyelid	Intermediate
C18	Correction of ptosis of eyelid	Intermediate
C22	Other operations on eyelid	Intermediate
C25	Connection between lacrimal apparatus and nose	Intermediate
C27	Operations on nasolacrimal duct	Intermediate
C29	Other operations on lacrimal apparatus	Intermediate
C31	Combined operations on muscles of eye	Intermediate
C32	Recession of muscle of eye	Intermediate
C39	Extirpation of lesion of conjunctiva	Intermediate
C44	Other plastic operations on cornea	Intermediate
C45	Extirpation of lesion of cornea	Intermediate
C46	Plastic operations on cornea	Intermediate
C47	Closure of cornea	Intermediate

Procedure Code	Procedure Description	Abbott Category
C51	Other operations on cornea	Intermediate
C60	Filtering operations on iris	Intermediate
C61	Other operations on trabecular meshwork of eye	Intermediate
C62	Incision of iris	Intermediate
C65	Operations following glaucoma surgery	Intermediate
C66	Extirpation of ciliary body	Intermediate
C71	Extracapsular extraction of lens	Intermediate
C73	Incision of capsule of lens	Intermediate
C75	Prosthesis of lens	Intermediate
C79	Operations on vitreous body	Intermediate
C82	Destruction of lesion of retina	Intermediate
C85	Fixation of retina	Intermediate
C86	Other operations on eye	Intermediate
C89	Operations on posterior segment of eye	Intermediate
D01	Excision of external ear	Intermediate
D02	Extirpation of lesion of external ear	Intermediate
D03	Plastic operations on external ear	Intermediate
D06	Other operations on external ear	Intermediate
D13	Attachment of bone anchored hearing prosthesis	Intermediate
D14	Repair of eardrum	Intermediate
D15	Drainage of middle ear	Intermediate
D20	Other operations on middle ear	Intermediate
D24	Operations on cochlea	Intermediate
D28	Other operations on ear	Intermediate
E02	Plastic operations on nose	Intermediate
E03	Operations on septum of nose	Intermediate
E04	Operations on turbinate of nose	Intermediate
E05	Surgical arrest of bleeding from internal nose	Intermediate
E07	Other plastic operations on nose	Intermediate
E08	Other operations on internal nose	Intermediate
E09	Operations on external nose	Intermediate
E14	Operations on frontal sinus	Intermediate
E20	Operations on adenoid	Intermediate
E34	Microtherapeutic endoscopic operations on larynx	Intermediate
E35	Other therapeutic endoscopic operations on larynx	Intermediate
E63	Diagnostic endoscopic examination of mediastinum	Intermediate
F02	Extirpation of lesion of lip	Intermediate
F05	Other repair of lip	Intermediate
F06	Other operations on lip	Intermediate
F09	Surgical removal of tooth	Intermediate
F18	Excision of dental lesion of jaw	Intermediate
F24	Incision of tongue	Intermediate
F26	Other operations on tongue	Intermediate

Procedure Code	Procedure Description	Abbott Category
F32	Other operations on palate	Intermediate
F34	Excision of tonsil	Intermediate
F36	Other operations on tonsil	Intermediate
F42	Other operations on mouth	Intermediate
F44	Excision of salivary gland	Intermediate
F48	Other operations on salivary gland	Intermediate
G75	Attention to artificial opening into ileum	Intermediate
H44	Manipulation of rectum	Intermediate
H48	Excision of lesion of anus	Intermediate
H51	Excision of haemorrhoid	Intermediate
H52	Destruction of haemorrhoid	Intermediate
H55	Other operations on perianal region	Intermediate
H56	Other operations on anus	Intermediate
H58	Drainage through perineal region	Intermediate
H59	Excision of pilonidal sinus	Intermediate
H60	Other operations on pilonidal sinus	Intermediate
J10	Transluminal operations on blood vessel of liver	Intermediate
J12	Other therapeutic percutaneous operations on liver	Intermediate
J13	Diagnostic percutaneous operations on liver	Intermediate
K59	Cardioverter defibrillator introduced through the vein	Intermediate
K60	Cardiac pacemaker system introduced through vein	Intermediate
K62	Therapeutic transluminal operations on heart	Intermediate
K65	Catheterisation of heart	Intermediate
K73	Other cardiac pacemaker system introduced through vein	Intermediate
K75	Percutaneous transluminal balloon angioplasty and insertion of stent into coronary artery	Intermediate
L35	Transluminal operations on cerebral artery	Intermediate
L54	Transluminal operations on iliac artery	Intermediate
L63	Transluminal operations on femoral artery	Intermediate
L66	Other therapeutic transluminal operations on artery	Intermediate
L71	Therapeutic transluminal operations on other artery	Intermediate
L84	Combined operations on varicose vein of leg	Intermediate
L86	Injection into varicose vein of leg	Intermediate
L87	Other operations on varicose vein of leg	Intermediate
L88	Transluminal operations on varicose vein of leg	Intermediate
L91	Other vein related operations	Intermediate
L94	Therapeutic transluminal operations on vein	Intermediate
L99	Other therapeutic transluminal operations on vein	Intermediate
M09	Therapeutic endoscopic operations on calculus of kidney	Intermediate
M13	Percutaneous puncture of kidney	Intermediate
M16	Other operations on kidney	Intermediate
M27	Therapeutic ureteroscopic operations on ureter	Intermediate
M28	Other endoscopic removal of calculus from ureter	Intermediate

Procedure Code	Procedure Description	Abbott Category
M29	Other therapeutic endoscopic operations on ureter	Intermediate
M33	Percutaneous ureteric stent procedures	Intermediate
M38	Open drainage of bladder	Intermediate
M42	Endoscopic extirpation of lesion of bladder	Intermediate
M43	Endoscopic operations to increase capacity of bladder	Intermediate
M44	Other therapeutic endoscopic operations on bladder	Intermediate
M49	Other operations on bladder	Intermediate
M56	Therapeutic endoscopic operations on outlet of female bladder	Intermediate
M65	Endoscopic resection of outlet of male bladder	Intermediate
M66	Other therapeutic endoscopic operations on outlet of male bladder	Intermediate
M70	Other operations on outlet of male bladder	Intermediate
M76	Therapeutic endoscopic operations on urethra	Intermediate
M79	Other operations on urethra	Intermediate
M81	Operations on urethral orifice	Intermediate
N01	Extirpation of scrotum	Intermediate
N03	Other operations on scrotum	Intermediate
N07	Extirpation of lesion of testis	Intermediate
N08	Bilateral placement of testes in scrotum	Intermediate
N09	Other placement of testis in scrotum	Intermediate
N11	Operations on hydrocele sac	Intermediate
N13	Other operations on testis	Intermediate
N15	Operations on epididymis	Intermediate
N17	Excision of vas deferens	Intermediate
N19	Operations on varicocele	Intermediate
N27	Extirpation of lesion of penis	Intermediate
N28	Plastic operations on penis	Intermediate
N30	Operations on prepuce	Intermediate
N32	Other operations on penis	Intermediate
O01	Transluminal coil embolisation of aneurysm of artery	Intermediate
P03	Operations on Bartholin gland	Intermediate
P06	Extirpation of lesion of vulva	Intermediate
P09	Other operations on vulva	Intermediate
P13	Other operations on female perineum	Intermediate
P23	Other repair of prolapse of vagina	Intermediate
P29	Other operations on vagina	Intermediate
Q02	Destruction of lesion of cervix uteri	Intermediate
Q09	Other open operations on uterus	Intermediate
Q10	Curettage of uterus	Intermediate
Q11	Other evacuation of contents of uterus	Intermediate
Q16	Other vaginal operations on uterus	Intermediate
Q17	Therapeutic endoscopic operations on uterus	Intermediate
Q20	Other operations on uterus	Intermediate
Q35	Endoscopic bilateral occlusion of fallopian tubes	Intermediate

Procedure Code	Procedure Description	Abbott Category
Q41	Other operations on fallopian tube	Intermediate
Q49	Therapeutic endoscopic operations on ovary	Intermediate
Q54	Operations on other ligament of uterus	Intermediate
R12	Operations on gravid uterus	Intermediate
R17	Elective caesarean delivery	Intermediate
R18	Other caesarean delivery	Intermediate
S05	Microscopically controlled excision of lesion of skin	Intermediate
S06	Other excision of lesion of skin	Intermediate
S35	Split autograft of skin	Intermediate
S41	Suture of skin of head or neck	Intermediate
S42	Suture of skin of other site	Intermediate
S47	Opening of skin	Intermediate
S56	Exploration of other skin of head or neck	Intermediate
S57	Exploration of other skin of other site	Intermediate
S60	Other operations on skin	Intermediate
S62	Other operations on subcutaneous tissue	Intermediate
S64	Extirpation of nail bed	Intermediate
S66	Other operations on nail bed	Intermediate
S68	Excision of nail	Intermediate
S70	Other operations on nail	Intermediate
T12	Puncture of pleura	Intermediate
T19	Simple excision of inguinal hernial sac	Intermediate
T20	Primary repair of inguinal hernia	Intermediate
T21	Repair of recurrent inguinal hernia	Intermediate
T22	Primary repair of femoral hernia	Intermediate
T24	Primary repair of umbilical hernia	Intermediate
T25	Primary repair of incisional hernia	Intermediate
T26	Repair of recurrent incisional hernia	Intermediate
T27	Repair of other hernia of abdominal wall	Intermediate
T31	Other operations on anterior abdominal wall	Intermediate
T42	Therapeutic endoscopic operations on peritoneum	Intermediate
T43	Diagnostic endoscopic examination of peritoneum	Intermediate
T45	Image controlled operations on abdominal cavity	Intermediate
T54	Division of fascia	Intermediate
T57	Other operations on fascia	Intermediate
T59	Excision of ganglion	Intermediate
T62	Operations on bursa	Intermediate
T64	Transposition of tendon	Intermediate
T67	Primary repair of tendon	Intermediate
T69	Freeing of tendon	Intermediate
T70	Adjustment to length of tendon	Intermediate
T72	Other operations on sheath of tendon	Intermediate
T74	Other operations on tendon	Intermediate

Procedure Code	Procedure Description	Abbott Category
T79	Repair of muscle	Intermediate
T87	Excision or biopsy of lymph node	Intermediate
T96	Other operations on soft tissue	Intermediate
T97	Repair of recurrent umbilical hernia	Intermediate
V21	Other operations on temporomandibular joint	Intermediate
W03	Complex reconstruction of forefoot	Intermediate
W04	Complex reconstruction of hindfoot	Intermediate
W12	Angulation periarticular division of bone	Intermediate
W13	Other periarticular division of bone	Intermediate
W26	Other closed reduction of fracture of bone	Intermediate
W33	Other open operations on bone	Intermediate
W59	Fusion of joint of toe	Intermediate
W69	Open operations on synovial membrane of joint	Intermediate
W71	Other open operations on intra-articular structure	Intermediate
W74	Other reconstruction of ligament	Intermediate
W75	Other open repair of ligament	Intermediate
W77	Stabilising operations on joint	Intermediate
W78	Release of contracture of joint	Intermediate
W79	Soft tissue operations on joint of toe	Intermediate
W81	Other open operations on joint	Intermediate
W82	Therapeutic endoscopic operations on semilunar cartilage	Intermediate
W83	Therapeutic endoscopic operations on other articular cartilage	Intermediate
W84	Therapeutic endoscopic operations on other joint structure	Intermediate
W85	Therapeutic endoscopic operations on cavity of knee joint	Intermediate
W87	Diagnostic endoscopic examination of knee joint	Intermediate
W89	Other therapeutic endoscopic operations on other articular cartilage	Intermediate
W91	Other manipulation of joint	Intermediate
W92	Other operations on joint	Intermediate
X11	Amputation of toe	Intermediate
A52	Therapeutic epidural injection	Inclusive
A54	Therapeutic spinal puncture	Inclusive
A55	Diagnostic spinal puncture	Inclusive
B32	Biopsy of breast	Inclusive
D07	Clearance of external auditory canal	Inclusive
E06	Packing of cavity of nose	Inclusive
E25	Diagnostic endoscopic examination of pharynx	Inclusive
E36	Diagnostic endoscopic examination of larynx	Inclusive
E37	Diagnostic microendoscopic examination of larynx	Inclusive
E49	Diagnostic fibreoptic endoscopic examination of lower respiratory tract	Inclusive
E65	Diagnostic endoscopic examination of nasal cavity	Inclusive
F10	Simple extraction of tooth	Inclusive
F12	Surgery on apex of tooth	Inclusive
F13	Restoration of tooth	Inclusive

Procedure Code	Procedure Description	Abbott Category
F14	Orthodontic operations	Inclusive
F16	Other operations on tooth	Inclusive
F20	Operations on gingiva	Inclusive
G15	Other therapeutic fibreoptic endoscopic operations on oesophagus	Inclusive
G16	Diagnostic fibreoptic endoscopic examination of oesophagus	Inclusive
G42	Other fibreoptic endoscopic extirpation of lesion of upper gastrointestinal tract	Inclusive
G43	Fibreoptic endoscopic extirpation of lesion of upper gastrointestinal tract	Inclusive
G44	Other therapeutic fibreoptic endoscopic operations on upper gastrointestinal tract	Inclusive
G45	Diagnostic fibreoptic endoscopic examination of upper gastrointestinal tract	Inclusive
G47	Intubation of stomach	Inclusive
G80	Diagnostic endoscopic examination of ileum	Inclusive
H20	Endoscopic extirpation of lesion of colon	Inclusive
H22	Diagnostic endoscopic examination of colon	Inclusive
H23	Endoscopic extirpation of lesion of lower bowel using fibreoptic sigmoidoscope	Inclusive
H24	Other therapeutic endoscopic operations on lower bowel using fibreoptic sigmoidoscope	Inclusive
H25	Diagnostic endoscopic examination of lower bowel using fibreoptic sigmoidoscope	Inclusive
J38	Endoscopic incision of sphincter of Oddi	Inclusive
J40	Endoscopic retrograde placement of prosthesis in bile duct	Inclusive
J41	Other therapeutic endoscopic retrograde operations on bile duct	Inclusive
J43	Diagnostic endoscopic retrograde examination of bile duct and pancreatic duct	Inclusive
J53	Endoscopic ultrasound examination of bile duct	Inclusive
J74	Endoscopic ultrasound examination of pancreas	Inclusive
K49	Transluminal balloon angioplasty of coronary artery	Inclusive
K58	Diagnostic transluminal operations on heart	Inclusive
K61	Other cardiac pacemaker system	Inclusive
L95	Diagnostic transluminal operations on vein	Inclusive
M14	Extracorporeal fragmentation of calculus of kidney	Inclusive
M30	Diagnostic endoscopic examination of ureter	Inclusive
M31	Extracorporeal fragmentation of calculus of ureter	Inclusive
M45	Diagnostic endoscopic examination of bladder	Inclusive
M77	Diagnostic endoscopic examination of urethra	Inclusive
Q03	Biopsy of cervix uteri	Inclusive
Q12	Intrauterine contraceptive device	Inclusive
Q13	Introduction of gametes into uterine cavity	Inclusive
Q14	Introduction of abortifacient into uterine cavity	Inclusive
Q18	Diagnostic endoscopic examination of uterus	Inclusive
Q48	Oocyte recovery	Inclusive

Procedure Code	Procedure Description	Abbott Category
Q55	Other examination of female genital tract	Inclusive
R14	Surgical induction of labour	Inclusive
R21	Forceps cephalic delivery	Inclusive
R22	Vacuum delivery	Inclusive
R32	Repair of obstetric laceration	Inclusive
S07	Photodynamic therapy of skin	Inclusive
S08	Curettage of lesion of skin	Inclusive
S09	Photodestruction of lesion of skin	Inclusive
S13	Punch biopsy of skin	Inclusive
S15	Other biopsy of skin	Inclusive
S43	Removal of repair material from skin	Inclusive
S44	Removal of other inorganic substance from skin	Inclusive
S45	Removal of other substance from skin	Inclusive
S53	Introduction of substance into skin	Inclusive
T46	Other drainage of peritoneal cavity	Inclusive
T81	Biopsy of muscle	Inclusive
W36	Diagnostic puncture of bone	Inclusive
W66	Primary closed reduction of traumatic dislocation of joint	Inclusive
W90	Puncture of joint	Inclusive
X41	Placement of ambulatory apparatus for compensation for renal failure	Inclusive
X59	Anaesthetic without surgery	Inclusive

Table S2. Search strategies used for targeted literature reviews for the 10 identified procedures

Cervical Decompression

1. neck muscles.sh.
2. exp Neck/
3. whiplash injuries.sh.
4. neck.ti,ab.
5. exp Cervical Vertebrae/
6. or/1-5
7. exp Spinal Cord Compression/
8. exp Spinal Osteophytosis/
9. exp Spinal Nerve Roots/
10. exp Radiculopathy/
11. radiculopathy.mp.
12. myelopathy.mp.
13. radiculomyelopathy.mp.
14. myeloradiculopathy.mp.
15. or/7-14
16. exp Surgery/
17. exp Surgical Procedures, Operative/
18. surgery.mp.
19. surgical.mp.
20. or/16-19
21. and/6,15,20

Deep Brain Stimulation

1. exp Deep Brain Stimulation/
2. (deep adj3 brain adj3 (stimulat* or stimuli* or stimulu*)).mp.
3. or/1-2

Hip Replacement

1. arthroplasty, replacement, hip/
2. Hip Prosthesis/
3. or/1-2
4. arthroplasty/ or arthroplasty, replacement/
5. Joint Prosthesis/
6. "Prostheses and Implants"/
7. (arthroplasty or replacement or prosthes#s).tw.
8. or/4-7
9. hip/ or hip joint/ or hip.tw.
10. 8 and 9
11. 3 or 10

Knee Replacement

1. Arthritis/

2. arthrit*.tw.
3. osteoarthr*.tw.
4. gonarthrosis.tw.
5. arthralgia.tw.
6. felty* syndrome.tw.
7. caplan* syndrome.tw.
8. sjogren* syndrome.tw.
9. sicca syndrome.tw.
10. still* disease.tw.
11. bechterew* disease.tw.
12. rheuma*.tw.
13. or/1-12
14. knee/
15. knee joint/
16. knee*.tw.
17. or/14-16
18. 13 and 17
19. knee arthroplasty/
20. knee prosthesis/
21. (knee* adj3 (arthroplast* or implant* or replace* or prosthe* or endoprosthe*)).tw.
22. or/19-21
23. "Prostheses and Implants"/
24. 17 and 23
25. 22 or 24
26. cementation/
27. "Bone cements"/
28. "Durapatite"/
29. (cement* or uncement* or Hydroxyapatite or Durapatite or Ingrowth or hybrid or porous* or coat* or press$fit).tw.
30. or/26-29
31. 18 and 25 and 30

Obesity Surgery

1. exp obesity/
2. Overweight/
3. over?weight.ti,ab.
4. over weight.ti,ab.
5. overeating.ti,ab.
6. over?eating.ti,ab.
7. exp Weight Loss/
8. weight loss.ti,ab.
9. weight reduc$.ti,ab.
10. or/1-9
11. bariatric surg$.ti,ab.
12. exp bariatric surgery/
13. (surg$ adj5 bariatric).ti,ab.
14. anti?obesity surg$.ti,ab.
15. antiobesity surg$.ti,ab.
16. (obesity adj5 surgery).ti,ab.

17. (obesity adj5 surgical).ti,ab.
18. (gastroplasty or gastro?gastostomy or "gastric bypass" or "gastric surgery" or "restrictive surgery").ti,ab.
19. exp gastric bypass/
20. exp jejunoileal bypass/
21. jejuno?ileal bypass.ti,ab.
22. jejunoileal bypass.ti,ab.
23. gastrointestinal surg$.ti,ab.
24. gastrointestinal diversion$.ti,ab.
25. exp biliopancreatic diversion/
26. biliopancreatic diversion.ti,ab.
27. bilio?pancreatic diversion.ti,ab.
28. biliopancreatic bypass.ti,ab.
29. bilio?pancreatic bypass.ti,ab.
30. gastric band$.ti,ab.
31. silicon band$.ti,ab.
32. exp gastroenterostomy/
33. gastrectomy.ti,ab.
34. gastrectomy.ti,ab.
35. gastroplasty/
36. LAGB.ti,ab.
37. stomach stapl$.ti,ab.
38. lap band$.ti,ab.
39. lap$band$.ti,ab.
40. malabsorptive surg$.ti,ab.
41. mason$ procedure.ti,ab.
42. "Roux-en-Y".ti,ab.
43. Anastomosis, Roux-en-Y/
44. malabsorptive procedure$.ti,ab.
45. duodenal switch$.ti,ab.
46. stomach stapl$.ti,ab.
47. obesity/su
48. exp Obesity, Morbid/su [Surgery]
49. or/11-48
50. and/10,49

Prostatectomy

1. exp Prostatic Neoplasms/
2. exp Prostatic Intraepithelial Neoplasia/
3. (prostat* adj3 (cancer* or carcinoma* or malignan* or tumo?r* or neoplas* or adeno* or intraepithelial)).tw.
4. exp prostate/
5. or/1-4
6. exp Prostatectomy/
7. prostatectom*.tw.
8. (prostat* adj3 (remov* or resect*)).tw.
9. prostate/su
10. (excis* adj3 prostat*).tw.
11. or/6-10

12. and/5,11

Radiofrequency Denervation

1. dorsalgia.ti,ab.
2. exp Back Pain/
3. backache.ti,ab.
4. (lumbar adj pain).ti,ab.
5. coccyx.ti,ab.
6. coccydynia.ti,ab.
7. sciatica.ti,ab.
8. sciatic neuropathy/
9. spondylosis.ti,ab.
10. lumbago.ti,ab.
11. or/1-10
12. exp Spine/
13. discitis.ti,ab.
14. exp Spinal Diseases/
15. (disc adj degeneration).ti,ab.
16. (disc adj prolapse).ti,ab.
17. (disc adj herniation).ti,ab.
18. spinal fusion.sh.
19. (facet adj joints).ti,ab.
20. intervertebral disc.sh.
21. postlaminectomy.ti,ab.
22. arachnoiditis.ti,ab.
23. (failed adj back).ti,ab.
24. or/12-23
25. 11 or 24
26. exp Radio Waves/
27. exp Pulsed Radiofrequency Treatment/
28. radiofrequency.mp.
29. radio frequency.mp.
30. exp Electrocoagulation/
31. electrocoag$.mp.
32. thermocoag$.mp.
33. (neurotom$ or neuroly$).mp.
34. or/26-33
35. 25 and 34

Shoulder Replacement

1. exp osteoarthritis/
2. osteoarthr$.tw.
3. (degenerative adj2 arthritis).tw.
4. arthrosis.tw.
5. arthropat$.tw.
6. rotator cuff arthro$.tw.
7. or/1-6
8. Shoulder/

9. shoulder joint/
10. shoulder pain/
11. shoulder$.tw.
12. or/8-11
13. (arthroplast$ or hemiarthroplast$ or (joint$ adj2 replace$)).tw.
14. (surface$ adj replace$).tw.
15. Arthroplasty, Replacement, Shoulder/
16. resurfac$.tw.
17. RTSA.tw.
18. glenoid.tw.
19. glenosphere.tw.
20. exp "Prostheses and Implants"/
21. (glenoid adj2 component).tw.
22. (humor$ adj2 component).tw.
23. endopro$.tw.
24. reverse.tw.
25. or/13-24
26. 7 and 12 and 25

Traumatic Dislocation

1. Shoulder Dislocation/
2. exp Joint Dislocations/
3. (shoulder$ adj3 (dislocat* or sublux* or instability or unstable)).tw.
4. (ankle$ adj3 (dislocat$ or sublux$ or instability or unstable)).tw.
5. (glenohumeral adj (joint or instability or unstable)).tw.
6. (lesion$1 adj (Hill Sachs or Bankart)).tw.
7. hill sachs lesion.tw.
8. or/1-7
9. Bankart.tw.
10. (arthroscop* or repair* or stabilis* or stabiliz* or reduc*).tw.
11. exp Closed Fracture Reduction/ or exp Open Fracture Reduction/
12. or/9-11
13. 8 and 12

Table S3. The most common primary procedures and primary diagnoses recorded for each of the 10 included 'restrictive' procedures.

Procedure code and description		Primary Procedure (% with this primary procedure)	Primary Diagnosis (% with this primary diagnosis)
		Procedures with high variation (SCV 2018/19)	
G32	Connection of stomach to transposed jejunum	Bypass of stomach by anastomosis of stomach to transposed jejunum (95%)	Obesity (92%)
V48	Denervation of spinal facet joint of vertebra	Radiofrequency controlled thermal denervation of spinal facet joint of lumbar vertebra (67%); Denervation of spinal facet joint of lumbar vertebra NEC (15%)	Dorsalgia (50%); Spondylosis (26%); Other intervertebral disc disorders (13%)
W41	Total prosthetic replacement of knee joint not using cement	Primary total prosthetic replacement of knee joint not using cement (93%)	Gonarthrosis [arthrosis of knee] (85%)
W93	Hybrid prosthetic replacement of hip joint using cemented acetabular component	Primary hybrid prosthetic replacement of hip joint using cemented acetabular component (92%)	Coxarthrosis [arthrosis of hip] (76%)
		Procedures with high growth (2014/15-2018/19)	
A09	Neurostimulation of brain	Maintenance of neurostimulator in brain (49%); Implantation of neurotransmitter into brain (23%); Other specified neurostimulation of brain (12%)	Parkinson's disease (42%); Fitting/Adjustment of other devices (15%); Dystonia (13%)
M61	Open excision of prostate	Total excision of prostate and capsule of prostate (83%)	Malignant neoplasm of prostate (97%)
V22	Primary decompression operations on cervical spine	Primary anterior decompression of cervical spinal cord and fusion of joint of cervical spine (49%); Other specified primary decompression operations on cervical spine (17%); Primary foraminotomy of cervical spine (11%)	Cervical disc disorders (42%); Other spondylopathies (14%); Spondylosis (13%)
W67	Secondary reduction of traumatic dislocation of joint	Secondary open reduction of fracture dislocation of joint and internal fixation NEC (63%); Remanipulation of traumatic dislocation of joint (15%); Remanipulation of fracture dislocation of joint (12%)	Fracture of lower leg, including ankle (52%)
W94	Hybrid prosthetic replacement of hip joint using cemented femoral	Primary hybrid prosthetic replacement of hip joint using cemented femoral component (95%)	Coxarthrosis [arthrosis of hip] (79%)

	component		
W97	Total prosthetic replacement of shoulder joint not using cement	Primary reverse polarity total prosthetic replacement of shoulder joint not using cement (74%); Primary total prosthetic replacement of shoulder joint not using cement (17%)	Other arthrosis (47%); Shoulder lesions (21%)

Table S4. Evidence of clinical and cost-effectiveness relating to highlighted procedures and related NICE guidance

Procedure (Code)	Evidence of effectiveness?	Evidence of cost-effectiveness?	NICE Guidance	Additional evidence
Procedures with high variation				
Bariatric surgical procedure (G32)	Yes from systematic reviews, with debate about procedure options[1, 2] and limited evidence in children and adolescents[3]	Yes compared to medical management [4-6]	People meeting specific criteria should be offered assessment for bariatric surgery CG189[7], QS127[8]	[9-11]
Procedure to alleviate pain in the spine (V48)	Not from systematic reviews.[12, 13] NICE reported that radiofrequency denervation is clinically effective at improving the pain score outcome for individuals that have severe low back pain.[14]	Limited; analysis undertaken by NICE indicated cost-effectiveness.[14 appendix N] A Dutch RCT found the procedure was not cost-effective.[15]	Evidence of cost-effectiveness was viewed as insufficient for a firm recommendation NG59[14]	[16]
Total knee replacement (uncemented) (W41)	Yes from a systematic review.[17]	Yes[18] though one study found that it was not cost-effective compared to partial knee replacement over a five-year period.[19]	People should be offered a choice of total or partial knee replacement as total knee replacement may be cost-effective over a longer than five-year follow-up period NG157.[20]	
Hip replacement (acetabular component) (W93)	Yes on the basis of observational data.[21]	No evidence of cost-effectiveness for this type of implant.[22]	'Prostheses for total hip replacement and resurfacing arthroplasty are recommended … only if the prostheses have rates (or projected rates) of revision of 5% or less at 10 years' TA304.[23]	

Procedure (Code)	Evidence of effectiveness?	Evidence of cost-effectiveness?	NICE Guidance	Additional evidence
Procedures with high growth				
Deep brain stimulation for involuntary posturing (A09)	Limited from a systematic review, with uncertain impact on quality of life.[24]	Mixed for Parkinson's disease. [25-27] Very limited for dystonia.[28, 29]	'Consider deep brain stimulation for people with advanced Parkinson's disease whose symptoms are not adequately controlled by best medical therapy' NG71.[30] For tremor and dystonia 'evidence … appears adequate to support the use of this procedure' IPG188.[31]	[32]
Open removal of prostate (M61)	Yes from a systematic review, although with potential drawbacks.[33]	Doubtful but may emerge with longer follow-up[34-37]	surgery or radiotherapy recommended for intermediate or high-risk prostate cancer NG131[38]; IPG193[39]	
Spine surgical procedure (V22)	Not from systematic reviews.[40, 41]	No evidence compared to medical management. One surgical procedure was found to be cost-effective compared to another.[42]	Refer people with axial spondyloarthritis to a complex spinal surgery service if two conditions apply NG65.[43]	[44, 45]
Joint surgical procedure (W67)	Not from a systematic review for ankles,[46] and limited for shoulders.[47] Trial evidence does not indicate effectiveness of surgical management of ankle or shoulder dislocation.[48-50]	No evidence compared to medical management.[50, 51]	Reported criterion for transferring people with a failed closed reduction of a hip joint to a specialist centre NG37.[52]	

Procedure (Code)	Evidence of effectiveness?	Evidence of cost-effectiveness?	NICE Guidance	Additional evidence
Hip replacement (femoral component) (W94)	Yes on the basis of observational data.[21]	No evidence of cost-effectiveness for this type of implant.[22]	'Prostheses for total hip replacement and resurfacing arthroplasty are recommended … only if the prostheses have rates (or projected rates) of revision of 5% or less at 10 years' TA304.[23]	
Shoulder replacement (W97)	Not from systematic reviews.[53-55]	No evidence compared to medical management.[56]	Unable to make recommendations for practice because of a lack of evidence NG157.[20]	

References

1. Colquitt JL, Pickett K, Loveman E, et al. Surgery for weight loss in adults. *Cochrane Db Syst Rev* 2014.
2. Picot J, Jones J, Colquitt JL, et al. Weight Loss Surgery for Mild to Moderate Obesity: A Systematic Review and Economic Evaluation. *Obes Surg* 2012; 22: 1496-1506.
3. Ells LJ, Mead E, Atkinson G, et al. Surgery for the treatment of obesity in children and adolescents (Review). *Cochrane Db Syst Rev* 2015.
4. Gulliford MC, Charlton J, Prevost T, et al. Costs and Outcomes of Increasing Access to Bariatric Surgery: Cohort Study and Cost-Effectiveness Analysis Using Electronic Health Records. *Value Health* 2017; 20: 85-92.
5. Tang Q, Sun ZP, Zhang NW, et al. Cost-Effectiveness of Bariatric Surgery for Type 2 Diabetes Mellitus A Randomized Controlled Trial in China. *Medicine* 2016; 95.
6. Pollock RF, Muduma G and Valentine WJ. Evaluating the cost-effectiveness of laparoscopic adjustable gastric banding versus standard medical management in obese patients with type 2 diabetes in the UK. *Diabetes Obes Metab* 2013; 15: 121-129.
7. National Institute for Health and Care Excellence. Obesity: identification, assessment and management (CG189), https://www.nice.org.uk/guidance/cg189 (2014).

8. National Institute for Health and Care Excellence. Obesity: clinical assessment and management (QS127), https://www.nice.org.uk/guidance/qs127 (2016).
9. Gounder ST, Wijayanayaka D, Murphy R, et al. Costs of bariatric surgery in a randomised control trial (RCT) comparing Roux en Y gastric bypass vs sleeve gastrectomy in morbidly obese diabetic patients. *New Zeal Med J* 2016; 129: 43-52.
10. Darabi S, Talebpour M, Zeinoddini A, et al. Laparoscopic gastric plication versus mini-gastric bypass surgery in the treatment of morbid obesity: A randomized clinical trial. *Surg Obes Relat Dis* 2013; 9: 914-919.
11. Rogers CA, Reeves BC, Byrne J, et al. Adaptation of the By-Band randomized clinical trial to By-Band-Sleeve to include a new intervention and maintain relevance of the study to practice. *Brit J Surg* 2017; 104: 1207-1214.
12. Maas ET, Ostelo RWJG, Niemisto L, et al. Radiofrequency denervation for chronic low back pain. *Cochrane Db Syst Rev* 2015.
13. Niemisto L, Kalso E, Malmivaara A, et al. Radiofrequency denervation for neck and back pain: A systematic review within the framework of the Cochrane Collaboration Back Review Group. *Spine* 2003; 28: 1877-1888.
14. National Institute for Health and Care Excellence. Low back pain and sciatica in over 16s: assessment and management (NG59), https://www.nice.org.uk/guidance/ng59 (2016).
15. Maas ET, Juch JNS, Ostelo RWJG, et al. Cost-Effectiveness of Radiofrequency Denervation for Patients With Chronic Low Back Pain: The MINT Randomized Clinical Trials. *Value Health* 2020; 23: 585-594.
16. Price C, Reeves B, Ahmad A, et al. Radiofrequency denervation of the lumbar facet joints: guidelines for the RADICAL randomised controlled trial. *Brit J Pain* 2020.
17. Nakama GY, Peccin MS, Almeida GJM, et al. Cemented, cementless or hybrid fixation options in total knee arthroplasty for osteoarthritis and other non-traumatic diseases. *Cochrane Db Syst Rev* 2012.
18. Dakin H, Gray A, Fitzpatrick R, et al. Rationing of total knee replacement: a cost-effectiveness analysis on a large trial data set. *Bmj Open* 2012; 2.
19. Beard DJ, Davies LJ, Cook JA, et al. The clinical and cost-effectiveness of total versus partial knee replacement in patients with medial compartment osteoarthritis (TOPKAT): 5-year outcomes of a randomised controlled trial. *Lancet* 2019; 394: 746-756.
20. National Institute for Health and Care Excellence. Joint replacement (primary): hip, knee and shoulder (NG157), www.nice.org.uk/guidance/ng157 (2020).
21. Fitzpatrick R SE, Sculpher M, et al. . Primary total hip replacement surgery: a systematic review of outcomes and modelling of cost-effectiveness associated with different prostheses. *Health Technol Assess* 1998; 2: 1–64.
22. Fawsitt C TH, Hunt L, et al. Choice of Prosthetic Implant Combinations in Total Hip Replacement: Cost-Effectiveness Analysis Using UK and Swedish Hip Joint Registries Data. *Value Health* 2019; 22: 303-312.
23. National Institute for Health and Care Excellence. Total hip replacement and resurfacing arthroplasty for end-stage arthritis of the hip (TA304), www.nice.org.uk/guidance/ta304 (2014).
24. Rodrigues FB, Duarte GS, Prescott D, et al. Deep brain stimulation for dystonia. *Cochrane Db Syst Rev* 2019.

25. McIntosh E, Gray A, Daniels J, et al. Cost-utility analysis of deep brain stimulation surgery plus best medical therapy versus best medical therapy in patients with Parkinson's: Economic evaluation alongside the PD SURG trial. *Movement Disord* 2016; 31: 1173-1182.
26. Stroupe KT, Weaver FM, Cao LS, et al. Cost of Deep Brain Stimulation for the Treatment of Parkinson's Disease by Surgical Stimulation Sites. *Movement Disord* 2014; 29: 1666-1674.
27. Eggington S, Valldeoriola F, Chaudhuri KR, et al. The cost-effectiveness of deep brain stimulation in combination with best medical therapy, versus best medical therapy alone, in advanced Parkinson's disease. *J Neurol* 2014; 261: 106-116.
28. Dang T RD, Connelly L. Cost-Effectiveness of Deep Brain Stimulation With Movement Disorders: A Systematic Review. *Mov Disord Clin Pract* 2019; 6: 348-358.
29. Yianni J GA, McIntosh E, et al. The costs and benefits of deep brain stimulation surgery for patients with dystonia: an initial exploration. *Neuromodulation* 2005; 8: 155–161.
30. National Institute for Health and Care Excellence. Parkinson’s disease in adults (NG71), www.nice.org.uk/guidance/ng71 (2017).
31. National Institute for Health and Care Excellence. Deep brain stimulation for tremor and dystonia (excluding Parkinson's disease) (IPG188), www.nice.org.uk/guidance/ipg188 (2006).
32. Williams A, Gill S, Varma T, et al. Deep brain stimulation plus best medical therapy versus best medical therapy alone for advanced Parkinson's disease (PD SURG trial): a randomised, open-label trial. *Lancet Neurol* 2010; 9: 581-591.
33. Vernooij RWM, Lancee M, Cleves A, et al. Radical prostatectomy versus deferred treatment for localised prostate cancer. *Cochrane Db Syst Rev* 2020.
34. Noble SM, Garfield K, Lane JA, et al. The ProtecT randomised trial cost-effectiveness analysis comparing active monitoring, surgery, or radiotherapy for prostate cancer. *Brit J Cancer* 2020; 123: 1063-1070.
35. Ramsay C, Pickard R, Robertson C, et al. Systematic review and economic modelling of the relative clinical benefit and cost-effectiveness of laparoscopic surgery and robotic surgery for removal of the prostate in men with localised prostate cancer. *Health Technol Assess* 2012; 16: 1-313. 2012/11/07.
36. Sharma V, Wymer KM, Borah BJ, et al. Cost-Effectiveness of Active Surveillance, Radical Prostatectomy and External Beam Radiotherapy for Localized Prostate Cancer: An Analysis of the ProtecT Trial. *J Urology* 2019; 202: 966-974.
37. Andersson SO, Andren O, Lyth J, et al. Managing localized prostate cancer by radical prostatectomy or watchful waiting: Cost analysis of a randomized trial (SPCG-4). *Scand J Urol Nephrol* 2011; 45: 177-183.
38. National Institute for Health and Care Excellence. Prostate cancer: diagnosis and management (NG131), www.nice.org.uk/guidance/ng131 (2019).
39. National Institute for Health and Care Excellence. Laparoscopic radical prostatectomy (IPG193), nice.org.uk/guidance/ipg193 (2006).
40. Nikolaidis I, Fouyas IP, Sandercock PAG, et al. Surgery for cervical radiculopathy or myelopathy. *Cochrane Db Syst Rev* 2010.
41. Jacobs W, Willems PC, van Limbeek J, et al. Single or double-level anterior interbody fusion techniques for cervical degenerative disc disease. *Cochrane Db Syst Rev* 2011.

42. Ament JD, Yang Z, Nunley P, et al. Cost-effectiveness of Cervical Total Disc Replacement vs Fusion for the Treatment of 2-Level Symptomatic Degenerative DiscDisease (vol 149, 1231, 2014). *Jama Surg* 2014; 149: 1295-1295.
43. National Institute for Health and Care Excellence. Spondyloarthritis in over 16s: diagnosis and management (NG65), https://www.nice.org.uk/guidance/NG65 (2017).
44. Kotter M, Kalsi-Ryan S, Milligan J, et al. FUSION 4 DCM: Posterior decompressive surgery with fixation for multi-level degenerative cervical myelopathy, https://fundingawards.nihr.ac.uk/award/NIHR131243 (2021-2027).
45. Thomson S AG, Selvanathan S, et al. . Posterior cervical foraminotomy versus anterior cervical discectomy for Cervical Brachialgia: the FORVAD RCT. *Health Technol Assess* 2023; 27: 1-228.
46. al. DCA-KHVMe. Surgical versus conservative interventions for treating ankle fractures in adults. *Cochrane Db Syst Rev* 2012.
47. Handoll HH, Almaiyah MA and Rangan A. Surgical versus non-surgical treatment for acute anterior shoulder dislocation. *Cochrane Database Syst Rev* 2004.
48. Keene DJ, Mistry D, Nam J, et al. The Ankle Injury Management (AIM) trial: a pragmatic, multicentre, equivalence randomised controlled trial and economic evaluation comparing close contact casting with open surgical reduction and internal fixation in the treatment of unstable ankle fractures in patients aged over 60 years. *Health Technol Assess* 2016; 20: 1-158. 2016/10/14.
49. Murray IR, Robinson PG, Goudie EB, et al. Open Reduction and Tunneled Suspensory Device Fixation Compared with Nonoperative Treatment for Type-III and Type-IV Acromioclavicular Joint Dislocations. *J Bone Joint Surg Am* 2018; 100: 1912-1918.
50. Slobogean GP, Marra CA, Sadatsafavi M, et al. Is Surgical Fixation for Stress-Positive Unstable Ankle Fractures Cost Effective? Results of a Multicenter Randomized Control Trial. *Journal of Orthopaedic Trauma* 2012; 26: 652-658. DOI: 10.1097/BOT.0b013e31824aec42.
51. White TO, Bugler KE, Appleton P, et al. A prospective randomised controlled trial of the fibular nail versus standard open reduction and internal fixation for fixation of ankle fractures in elderly patients. *Bone Joint J* 2016; 98b: 1248-1252.
52. National Institute for Health and Care Excellence. Fractures (complex): assessment and management (NG37), https://www.nice.org.uk/guidance/ng37 (2016).
53. Christie A, Dagfinrud H, Engen Matre K, et al. Surgical interventions for the rheumatoid shoulder. *Cochrane Db Syst Rev* 2010.
54. Craig RS, Goodier H, Singh JA, et al. Shoulder replacement surgery for osteoarthritis and rotator cuff tear arthropathy. *Cochrane Db Syst Rev* 2020.
55. Singh JA, Sperling J, Buchbinder R, et al. Surgery for shoulder osteoarthritis. *Cochrane Db Syst Rev* 2010.
56. Handoll H, Brealey S, Rangan A, et al. The ProFHER (PROximal Fracture of the Humerus: Evaluation by Randomisation) trial - a pragmatic multicentre randomised controlled trial evaluating the clinical effectiveness and cost-effectiveness of surgical compared with non-surgical treatment for proximal fracture of the humerus in adults. *Health Technol Asses* 2015; 19: 1-+.

Figure S1. Variation in standardised rates of connection of stomach to transposed jejunum (G32) by CCG in 2018/19

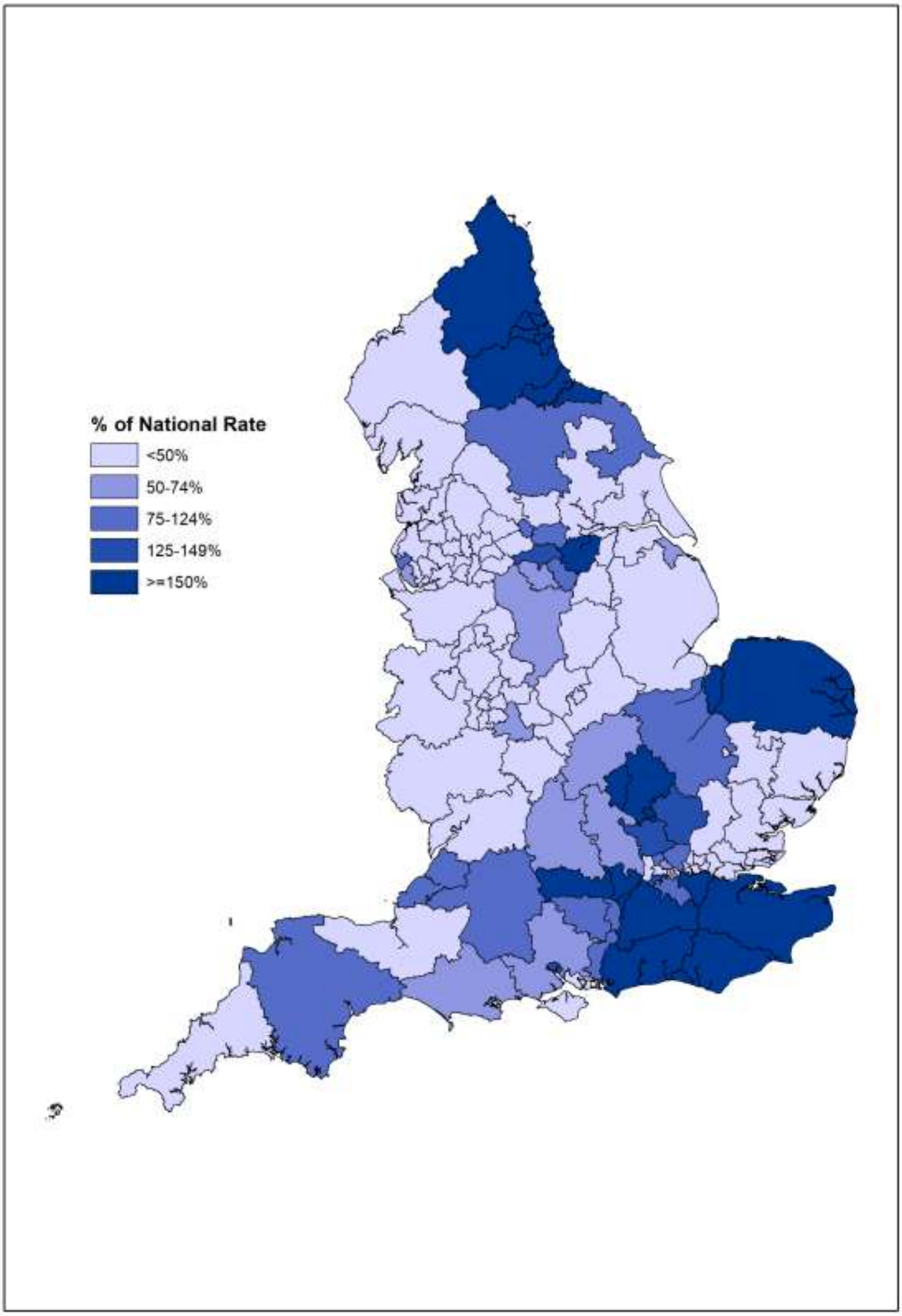

Figure S2. Variation in standardised rates of denervation of spinal facet joint of vertebra (V48) by CCG in 2018/19

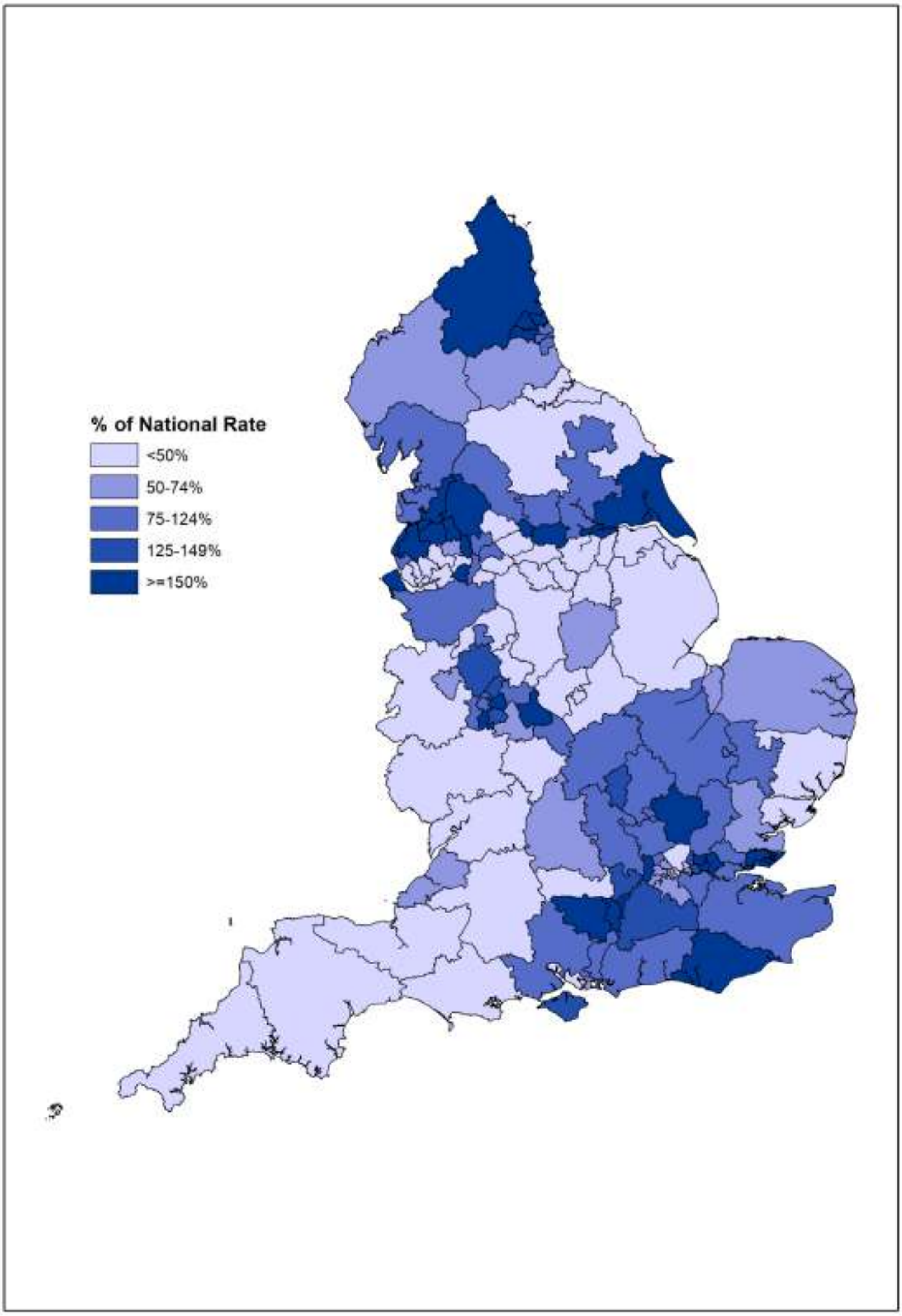

Figure S3. Variation in standardised rates of total prosthetic replacement of knee joint not using cement (W41) by CCG in 2018/19

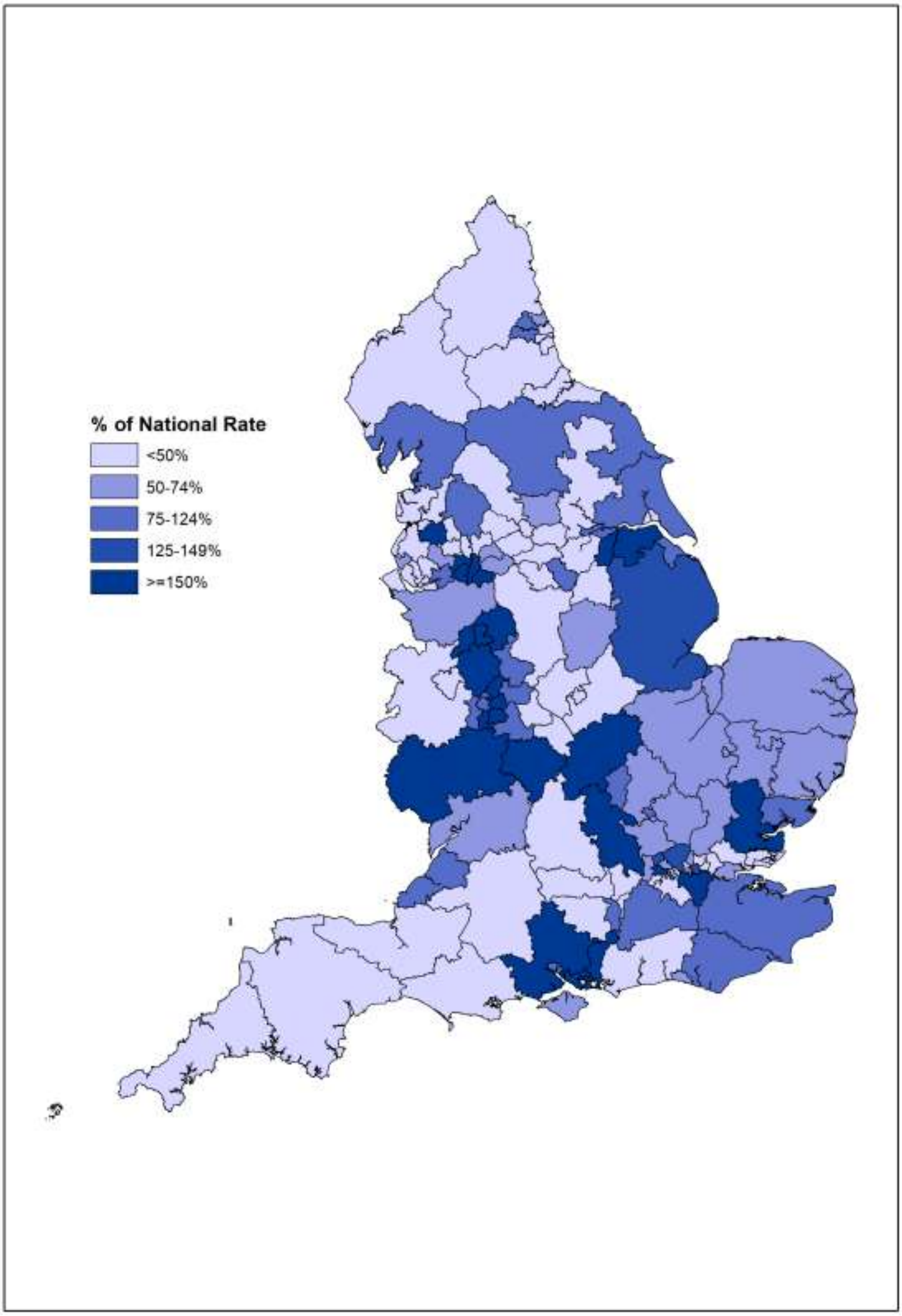

Figure S4. Variation in standardised rates of hybrid prosthetic replacement of hip joint using cemented acetabular component (W93) by CCG in 2018/19

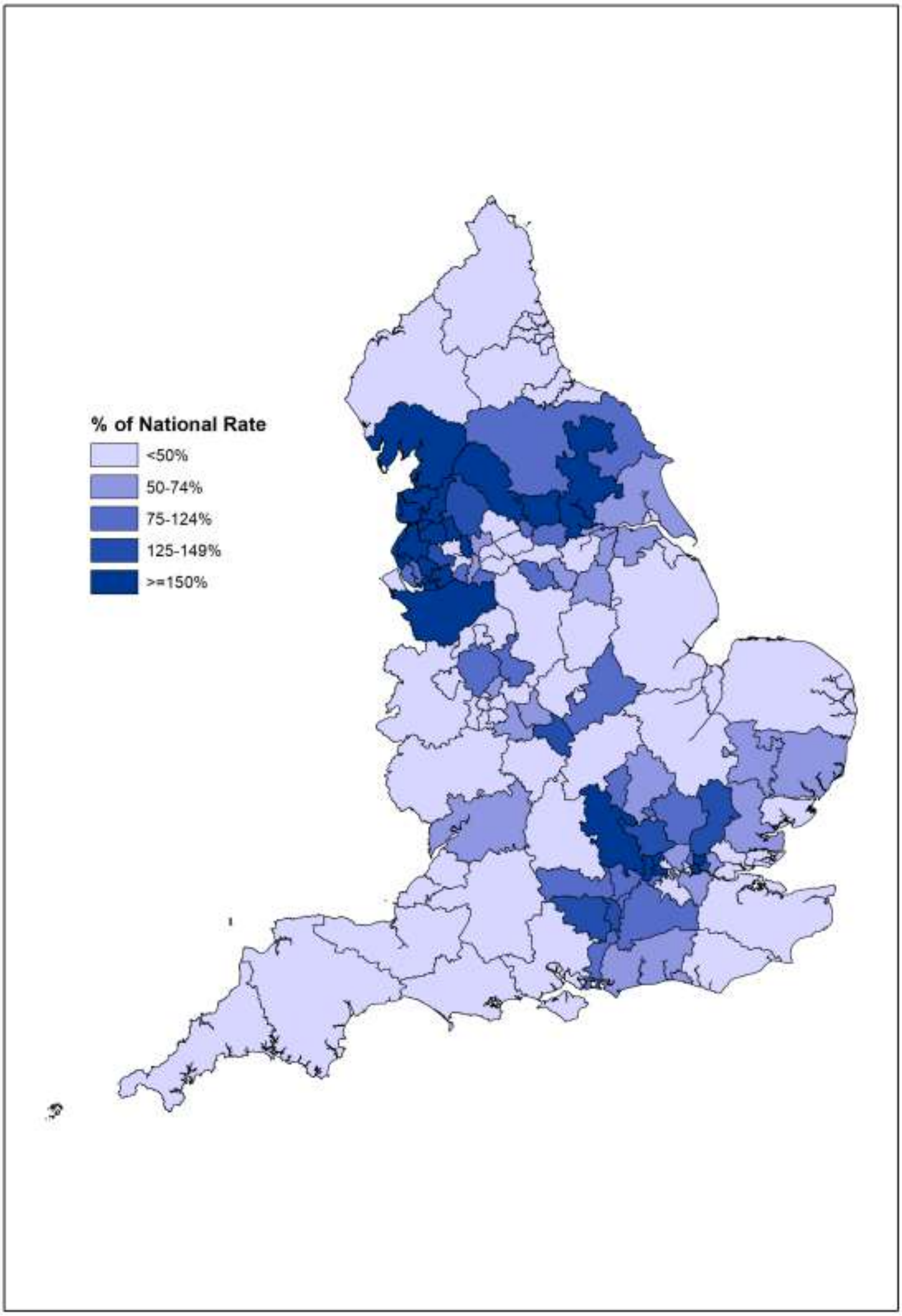

Figure S5. National procedure rates (2018/19), growth in procedure rates (2014/15-2018/19), and geographic variation in procedure rates (2018/19) for 'inclusive' procedures.

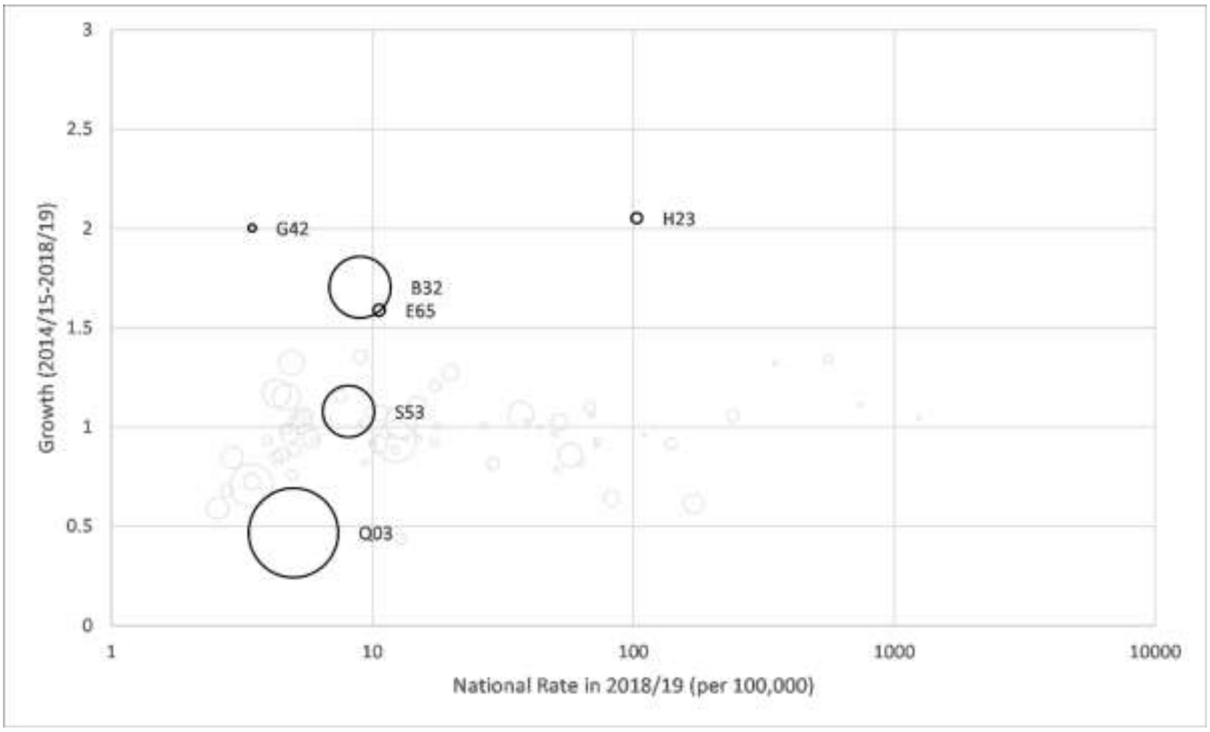

Note: Bubble size is proportional to systematic component of variance (2018/19). When growth is <1 the procedure rate has reduced over the 5 years. Highlighted entries are either top 5% in terms of growth (>1.59) or geographic variance in 2018/19 (>4.05). Procedure code labels are to the right of the corresponding circle. National rate in 2018/19 is represented on a log scale. G42 - Other fibreoptic endoscopic extirpation of lesion of upper gastrointestinal tract; H23 - Endoscopic extirpation of lesion of lower bowel using fibreoptic sigmoidoscope; B32 - Biopsy of breast; E65 - Diagnostic endoscopic examination of nasal cavity; S53 - Introduction of substance into skin; Q03 - Biopsy of cervix uteri. For B32, S53, and Q03, we are aware that variation is substantially reduced if inpatient and outpatient data are combined, suggesting there is some choice in which setting the procedures are carried out.

Figure S6. National procedure rates (2018/19), growth in procedure rates (2014/15-2018/19), and geographic variation in procedure rates (2018/19) for 'intermediate' procedures.

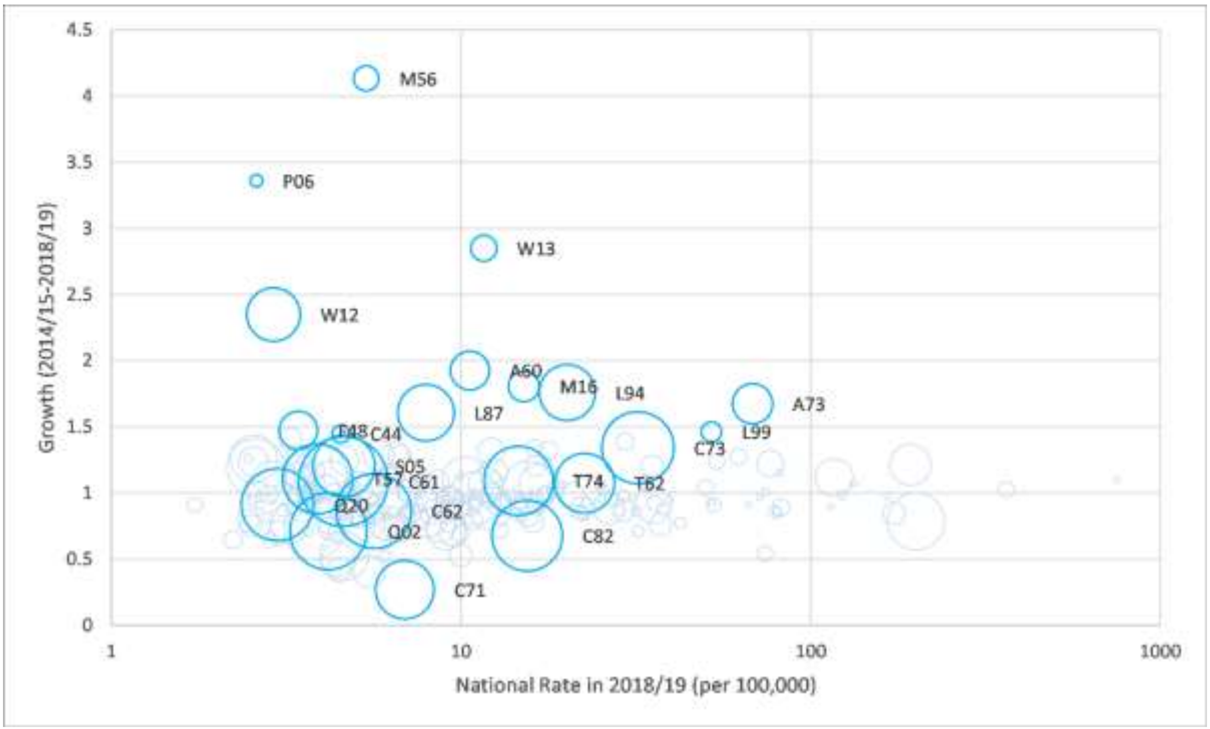

Note: Bubble size is proportional to systematic component of variance (2018/19). When growth is <1 the procedure rate has reduced over the 5 years. Highlighted entries are either the top 5% in terms of growth (>1.45) or geographic variance in 2018/19 (>1.79). Procedure code labels are to the right of the corresponding circle. National rate in 2018/19 is represented on a log scale. M56 - Therapeutic endoscopic operations on outlet of female bladder; P06 - Extirpation of lesion of vulva; W13 - Other periarticular division of bone; W12 - Angulation periarticular division of bone; A60 - Destruction of peripheral nerve; A73 - Other operations on peripheral nerve; C44 - Other plastic operations on cornea; C61 - Other operations on trabecular meshwork of eye; C62 - Incision of iris; C71 - Extracapsular extraction of lens; C73 - Incision of capsule of lens; C82 - Destruction of lesion of retina; F48 - Other operations on salivary gland; K73 - Other cardiac pacemaker system introduced through vein; L87 - Other operations on varicose vein of leg; L94 - Therapeutic transluminal operations on vein; L99 - Other therapeutic transluminal operations on vein; M16 - Other operations on kidney; Q02 - Destruction of lesion of cervix uteri; Q20 - Other operations on uterus; S05 - Microscopically controlled excision of lesion of skin; T57 - Other operations on fascia; T62 - Operations on bursa; T74 - Other operations on tendon

Methods S1. Systematic component of variance.

Equation 1. Formula for systematic component of variance.

$$SCV = \frac{\sum_i \frac{(o_i - e_i)^2}{e_i^2} - \sum_i \frac{1}{e_i^2}}{k}$$

, where o_i is the observed number of procedures in CCG *i*, e_i is the expected number of procedures in CCG *i*, and *k* is the number of CCGs. The SCV is equivalent to variance in the standardised incidence ratios with random variation removed, and is a measure of dispersion that takes into account the spread of all datapoints. It should not be affected by baseline counts/rates for a procedure, so can be compared across different procedures in the same region (e.g. England).[23] For additional information on geographical variation, we provided the standardised CCG procedure rate at the 95th percentile and the standardised CCG procedure rate at the 5th percentile (when ordered from lowest to highest).
